# Characterization of an envelope gene VP19 from Singapore grouper iridovirus

**DOI:** 10.1186/1743-422X-10-354

**Published:** 2013-12-16

**Authors:** Xiaohong Huang, Jie Gong, Youhua Huang, Zhengliang Ouyang, Shaowen Wang, Xiuli Chen, Qiwei Qin

**Affiliations:** 1 Key Laboratory of Tropical Marine Bio-resources and Ecology, South China Sea Institute of Oceanology, Chinese Academy of Sciences, 164 West Xingang Road, Guangzhou 510301, China; 2 State Key Laboratory of Biocontrol, School of Life Sciences, Sun Yat-sen University, 135 West Xingang Road, Guangzhou 510275, China

**Keywords:** SGIV, Iridovirus, VP19, Envelope protein, Virus assembly

## Abstract

**Background:**

Viral envelope proteins are always proposed to exert important function during virus infection and replication. Vertebrate iridoviruses are enveloped large DNA virus, which can cause great economic losses in aquaculture and ecological destruction. Although numerous iridovirus envelope proteins have been identified using bioinformatics and proteomic methods, their roles in virus infection remained largely unknown.

**Methods:**

Using SMART and TMHMM programs, we investigated the structural characteristics of Singapore grouper iridovirus (SGIV) VP19. A specific antibody against VP19 was generated and the expression profile of VP19 was clarified. The subcellular localization of VP19 in the absence or presence of other viral products was determined via transfection and immune fluorescence assay. In addition, Western blot assay and electron microscopy examination were performed to demonstrate whether SGIV VP19 was an envelope protein or a capsid protein.

**Results:**

Here, SGIV VP19 was cloned and characterized. Among all sequenced iridoviruses, VP19 and its orthologues shared common features, including 19 invariant cysteines, a proline-rich motif and a predicted transmembrane domain. Subsequently, the protein synthesis of VP19 was only detected at the late stage of SGIV infection and inhibited obviously by treating with AraC, confirming that VP19 was a late expressed protein. Ectopic expression of EGFP-VP19 *in vitro* displayed a punctate pattern in the cytoplasm. In SGIV infected cells, the newly synthesized VP19 protein was initially localized in the cytoplasm in a punctate pattern, and then aggregated into the virus assembly site at the late stage of SGIV infection, suggesting that other viral protein products were essential for VP19’s function during SGIV infection. In addition, Western blot assay and electron microscopy observation revealed that SGIV VP19 was associated with viral envelope, which was different from major capsid protein (MCP).

**Conclusion:**

Taken together, the current data suggested that VP19 represented a conserved envelope protein in iridovirus, and might contribute greatly to virus assembly during virus infection.

## Background

Iridoviruses are large, icosahedral, enveloped DNA viruses which can infect invertebrates and poikilothermic vertebrates. To date, the family *Iridovirade* was subdivided into five genera, including *Ranavirus, Megalocytivirus, Lymphocystivirus, Iridovirus* and *Chloriridovirus*[1,2]. Iridovirus infection and transmission among insect, fish, amphibians and reptiles causes great economic losses in aquaculture and ecological destruction [3-5]. To better understand the molecular mechanism of iridovirus pathogenesis and explore the strategy for prevention and control iridovirus diseases, complete genomes of 19 iridovirus isolates were sequenced and annotated up to now [6-8]. Some functional genes encoded by individual iridovirus, such as infection cell protein (ICP), capsid protein, membrane protein and virus replication associated enzymes have been identified and characterized [9-11]. However, the function of many iridovirus core structural genes remained largely unknown.

Singapore grouper iridovirus (SGIV), a novel ranavirus which belongs to family *Iridoviridae,* was isolated from the diseased grouper [3]. SGIV infection evoked enlarged spleen with haemorrhage in *vivo*, and induced non-apoptotic cell death in spleen cells in *vitro*[12,13]. Although the genome sequence, viral transcription program and global landscape of structural proteins of SGIV were investigated in recent years [14-17], the function of essential or core genes remained largely unknown. SGIV VP19, a conserved iridovirus core gene, was identified as an envelope protein using mass spectrometry [14,17]. However, the detailed expression pattern and localization of VP19 during SGIV infection still remained uncertain. Here, our results revealed that SGIV VP19 was a late gene and encoded a cytoplasmic protein associated with virus assembly. All these data will provide new insights into understanding the roles of envelope proteins in iridovirus pathogenesis.

## Results

### VP19 sequence was conserved among known iridovirus

**Figure 1:**
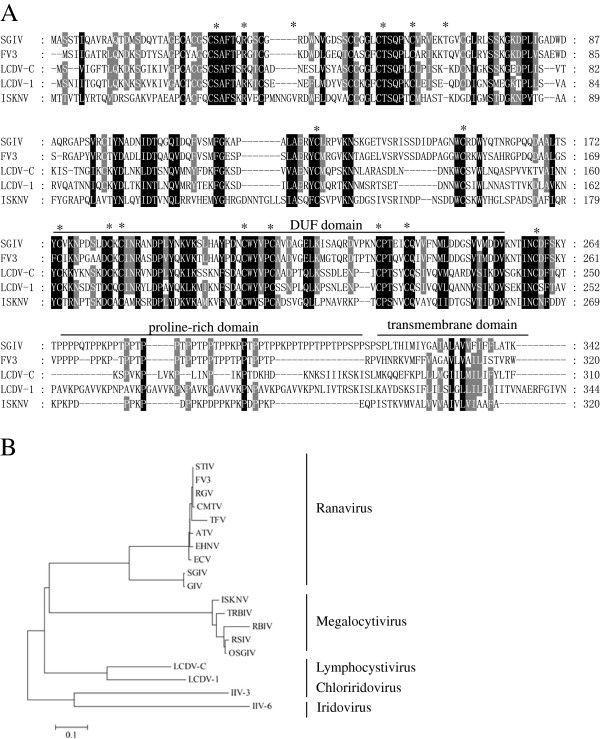
**Characteristics of SGIV VP19. (A)** Amino acid alignment of SGIV VP19 and other orthologues in family *Iridoviridae*. The black shaded regions indicate completely conserved residues, whilst the grey shaded regions are partially conserved residues with greater than 80% identity. The DUF domain was indicated with a line above the alignment. Conserved cysteines were highlighted with asterisks. The predicted transmembrane (TM) domain and the typical proline-rich motif were boxed with rectangle. **(B)** Phylogenetic position of VP19 in family *Iridoviridae*. Phylogenetic tree was constructed using neighbor-joining (NJ) method. Branch lengths are proportional to the evolutionary distance between the taxa.

SGIV VP19 was composed of 1029 bp and encoded a peptide of 342 aa with a predicted molecular weight of 36.7 kDa. In spite of a unique homolog existing in each sequenced iridoviruses, no significant sequence similarity between VP19 and any non-iridovirus proteins was found in the current database. Amino acid alignment indicated that VP19 contained a conserved DUF domain, 19 invariant cysteines potentially involved in disulfide bond formation and a carboxy-terminal transmembrane domain (Figure 1A). Interestingly, both VP19 and its orthologues shared a characteristic proline-rich motif which was also present in other viruses and proposed to play important roles in different aspects of the viral life cycle, including virus budding and release [18-20]. For better understanding of the SGIV VP19 position in evolutionary process, phylogenetic tree was constructed using neighbor-joining (NJ) method. As shown in Figure 1B, SGIV VP19 showed the closest relationship to GIV, and they made a subgroup in genus *Ranavirus*.

### The temporal expression pattern of VP19 during SGIV infection

**Figure 2:**
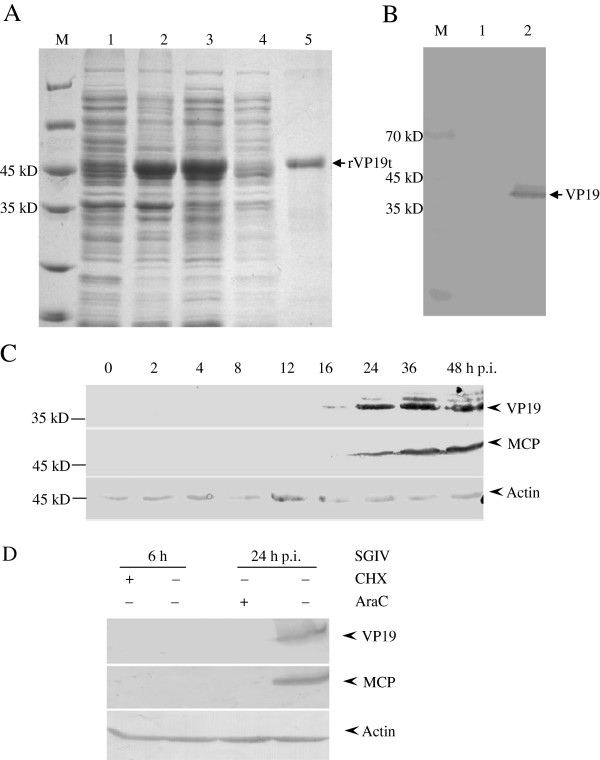
**Expression dynamics of SGIV VP19. (A)** SDS-PAGE analysis of purified recombinant SGIV VP19. Lines M, 1, 2, 3, 4 and 5 showed protein markers, pET-VP19 (uninduced), pET-VP19 (IPTG induced), supernatant, pellet of induced pET-VP19 and the purified pET-VP19, respectively. **(B)** The specificity of anti-VP19 serum. SGIV or mock infected cells at 48 h p.i. were collected and centrifuged for SDS-PAGE and western blotting. Line M, 1 and 2 showed protein markers, mock and SGIV infected cells at 48 h p.i., respectively. **(C)** The expression pattern of VP19 during SGIV infection. Actin was chosen as the internal control. **(D)** VP19 was identified as a late protein. Cells were infected with SGIV at MOI of 0.5 for 6 h and 24 h under the treatment with CHX or AraC, respectively. Then cells were collected for western blotting analysis. The late protein MCP was chosen as a positive control.

To obtain the anti-VP19 serum, the prokaryotic recombinant plasmid pET-VP19t was transformed into *E. coli* BL21 and induced by IPTG. As shown in Figure 2A, the recombinant fusion protein was observed in the supernatant of pET-VP19t after induction, but not in un-induced product. After purification, a single band at approximately 45 kD (fusion protein contained VP19t and His tag) was acquired and used to prepare anti-VP19 polyclonal antibody. The specificity of anti-VP19 antibody was examined using the lysates from mock-infected and SGIV infected GS cells at 24 h p.i. The results showed that the anti-VP19 antibody recognized the synthesized VP19 protein with molecular weight of 37 kD, while no protein band was detected in the mock-infected cell lysate (Figure 2B). In addition, no protein band was detected in the SGIV infected cell lysate when negative control serum was used as the primary antibody (data not shown).

To characterize the expression pattern of VP19 during SGIV infection, the protein synthesis level of VP19 at different time points were detected by western blotting. As shown in Figure 2C, VP19 specific protein band of ~37 kD was obviously detected from 24 h p.i., and its expression increased up to 48 h p.i. as well as the MCP specific band (~49 kD). As an internal control, the protein expression remained no obvious changes throughout SGIV infection. Further analysis using inhibitor assay showed that both VP19 and major capsid protein (MCP) expression could be significantly inhibited by the addition of AraC (Figure 2D), suggesting that VP19 was a late gene during SGIV infection.

### VP19 was localized in cytoplasm after transfection

**Figure 3:**
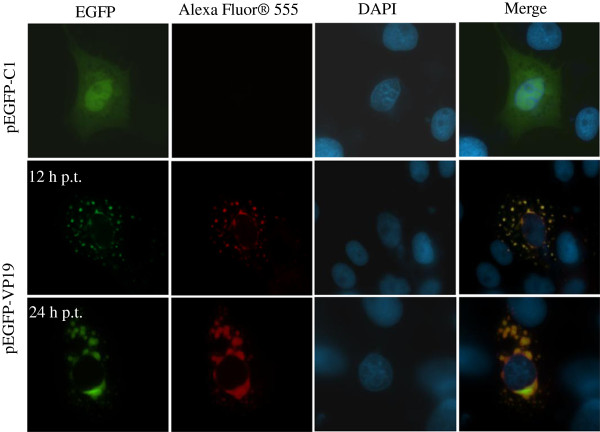
**Subcellular localization of VP19 in transfected cells.** After transfection with different plasmids, including pEGFP-C1 and EGFP-VP19, cells were fixed and the immune fluorescence assay were carried out using anti-VP19 primary antibody and Alexa Fluor® 555 Conjugated anti-mouse IgG. The green fluorescence indicated the distribution of EGFP-VP19 fusion protein, and the red fluorescence showed the VP19 protein recognized by antibody against VP19.

To clarify the localization of VP19 in *vitro*, we examined the green fluorescence in EGFP-VP19 transfected GS cells in the absence of other virus products. A punctate distribution of green fluorescence within cytoplasm was observed at 12 h post-transfection (p.t.). Both the sizes and density of fluorescence spots increased at 24 h p.t. Moreover, the overlapping fluorescence of EGFP (green fluorescence) and VP19 (red fluorescence) confirmed the correct expression of the full-length EGFP-VP19 fusion proteins *in vitro*. In contrast, no red fluorescence, but green fluorescence was observed in both the cytoplasm and nucleus in pEGFP-C1 transfected cells (Figure 3).

### VP19 aggregated into virus assembly sites at the late stage of SGIV infection

**Figure 4:**
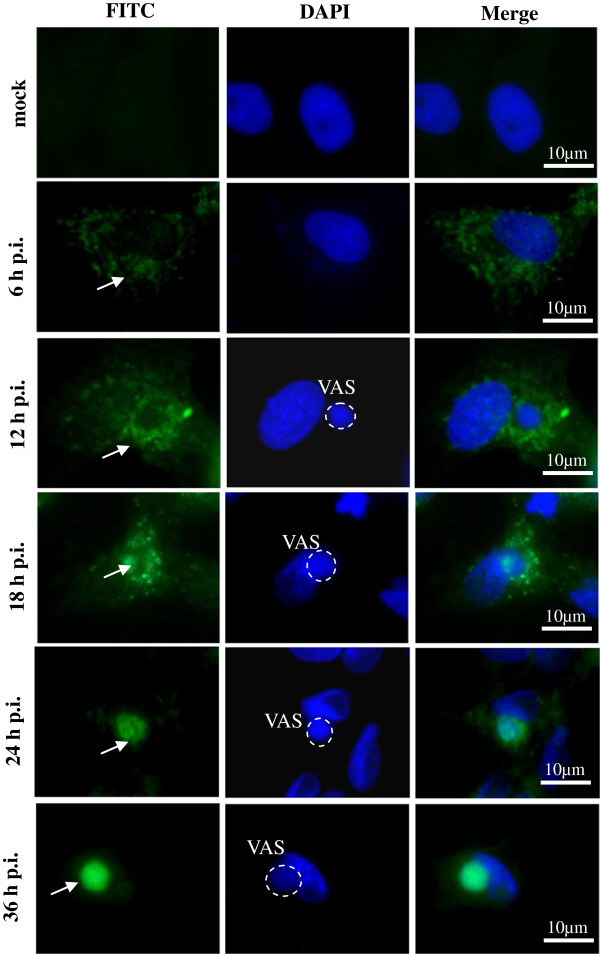
**SGIV VP19 aggregated into virus assembly sites at the late stage of SGIV infection.** Cells were infected with SGIV at MOI of 2, and then the immune fluorescence assay was carried out using antibody against VP19 at the indicated time points. Cell nuclei and viral DNA was stained with DAPI. The arrows showed the green fluorescence signal of VP19 protein, and the dashed circles indicated the virus assembly sites (VAS).

To demonstrate whether VP19 expression was influenced by other virus products, we detected the intracellular localization at indicated time points using anti-VP19 antibody. As shown in Figure 4, no green fluorescent signal was observed in mock infected cells. At 6 h p.i., faint punctate fluorescent spots were observed in the cytoplasm in a few cells. With the infection time increased, the numbers of the cells emitted green fluorescence and the fluorescence density increased. At 12 h p.i., the virus assembly sites could be observed, and the green fluorescence aggregated close to the virus assembly site. From 18 h p.i., the fluorescence signal began to appear in the virus assembly sites, and increased gradually. Until 36 h p.i., almost all the fluorescence signal aggregated into the virus assembly sites, suggesting that VP19 might participate in virus assembly with the help of other virus products.

### VP19 was confirmed as an envelope protein

**Figure 5:**
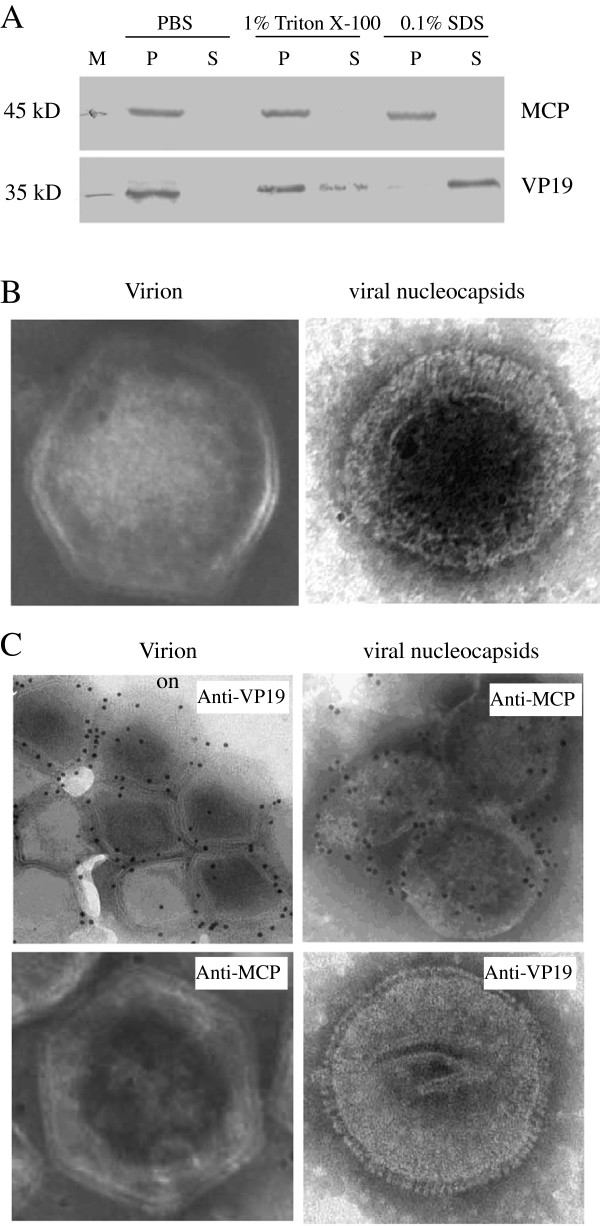
**VP19 was identified as an envelope protein. (A)** Western blotting revealed that VP19 could only be detected in the envelope fraction. Purified SGIV viral particles were incubated with PBS (pH 7.4), PBS with 1% Triton X-100, or PBS with 0.1% SDS separately as described in methods. After centrifugation, the supernatant (S) and pellet (P) fractions were electrophoresed by SDS-PAGE. Western blotting was performed using anti-VP19 or anti-MCP serum. **(B)** Ultrastructure of purified intact virus particles (virion) and viral nucleocapsids was determined under scan electron microscopy (SEM). **(C)** Ultrastructural localization of VP19 in virus particles. The localization of VP19 and MCP were detected after incubation with immunogold-labeled anti-VP19 and anti-MCP antibodies. The anti-VP19 antibody bound on virions, but not viral nucleocapsids, while the anti-MCP antibody bound on viral nucleocapsids, but not virions.

To ascertain whether VP19 was a component of envelope fraction of SGIV virus particles, purified virus from SGIV infected GS cells was treated with 1% Triton-X 100 as described previously [21]. As shown in Figure 5A, the majority of VP19 protein was detected in the pellets when treated with 1% Triton-X 100. After treating with other different detergents, including NP40, SDS and octyl-β-D-glucopyranoside (OG) (data not shown), we found that VP19 could be detected completely in the supernatant after treatment with 0.1% SDS. As a control, MCP protein was both detected in the pellets when the SGIV virus particles were treated with 1% Triton-X 100 or 0.1% SDS (Figure 5A). Efficient separation of VP19 from MCP indicated that SGIV VP19 was an envelope protein.

To further confirm the structure of VP19 in the SGIV virus particles, immuno-electron microscopy was carried out using anti-VP19 serum or anti-MCP antibody. As shown in Figure 5B, intact hexagon feature including outer envelope and capsid structure were observed in purified SGIV virus particles, while no envelope structure was observed in 0.1% SDS treated SGIV particles. Using anti-VP19 antibody, golden particles were observed on the surface of the intact virus particles, while no golden particles were observed on the intact virus particles. In contrast, using anti-MCP antibody, golden particles were observed only on 0.1% SDS treated virus particles, but not on the surface of the intact virus particles (Figure 5C). Together, the current results from Western blot assay and electron microscopy observation consistently confirmed that VP19 was an envelope protein.

## Discussion

Enveloped viruses usually initiated infection by membrane fusion between the virus and host cells. Increased reports revealed that virus encoded transmembrane (TM) domain contained proteins were always involved in this process [22,23]. Among the TM contained proteins encoded by SGIV, VP19 was one of the core genes present in all sequence iridoviruses. To date, homologs of VP19 could not be identified in non-iridovirus. The predictive TM domain at C-terminal might play crucial roles in its accurate subcellular localization or the formation of viral envelope [24,25]. The conserved cysteines in VP19 were proposed to participate into disulfide bond formation. For vaccinia virus A16 protein, the conserved cysteine residues were disulfide bonded via the poxvirus cytoplasmic redox system. A16 protein was further confirmed to be required for entry of poxviruses into cells as well as for cell-cell fusion [26]. Moreover, VP19 contained a DUF230 domain which was also found in vaccinia virus membrane protein A16 and G9 [27]. In addition, a typical proline-rich motif was found in VP19 and its homologs from iridovirus. Proline-rich motif in viral membrane proteins were confirmed to be involved in virus budding, release, and pathogenicity in *vivo*[19,20,28]. All these features co-existed in VP19, suggesting that VP19 might play important roles during SGIV pathogenesis. Other iridovirus envelope proteins encoded by RGV ORF53R and SGIV ORF088 have been characterized to exert vital roles in virus infection and assembly [10,17,29]. Therefore, our studies on VP19 will provide new insights into understanding the roles of envelope protein in iridovirus pathogenesis.

Increasing reports were focused on viral membrane proteins because of their complex and multiple roles during the various stages of virus infection, especially for large DNA viruses. Moreover, different detergents were attempted to extract the envelope proteins for different viruses [10,30,31]. To divide the SGIV virus particles into the envelope and capsid fraction effectively, a variety of detergents were used, including Triton X-100, NP40, octyl-β-D-glucopyranoside (OG) and SDS. Our results revealed that 1% Triton X-100 as well as NP40 or OG could not separate envelope from capsid fraction completely (data not shown). In contrast, both electron microscopy observation and Western blot assay indicated that treatment with 0.1% SDS resulted in complete removal of the envelope from intact virus particles like *Acidianus* filamentous virus 1 (AFV1) [30]. Using this method, we found that VP19 was only present in the envelope fraction, but not in the capsid fraction. Moreover, the reported envelope protein VP88 and capsid protein VP38 were also verified via treating with 0.1% SDS (unpublished data). Similar to VP19, another iridovirus core envelope protein RGV 53R has been reported to be associated with virus factory and involved in virion assembly [10,32]. In addition, immuno-electron microscopy studies showed that gold particles conjugated with anti-VP19 were only distributed on the surface of the virus envelope, while those conjugated with anti-MCP were only detected on the surface of capsid structure. Together, the current data confirmed that VP19 represented a conserved envelope protein existed in family *Iridoviridae*.

During DNA virus infection, some membrane proteins localized in virus assembly sites which can be stained by DAPI, while others instead accumulated in the specific compartments in cytoplasm ([33-35]. Moreover, recruitment of virion membrane proteins from the cytoplasm to the virus assembly sites could be mediated by the expression of other viral genes [35]. In our study, SGIV VP19 was localized in a punctate pattern within the cytoplasm in the absence of other virus products. Moreover, the size of the fluorescence spots increased with the time post-transfection. Of note, the synthesized VP19 could be detected in minority of the cells and displayed a punctate fluorescent pattern in the cytoplasm at 6 h p.i., and then aggregated into the virus assembly sites at the late stage of SGIV infection. The vaccinia virus envelope protein F13L protein expression exhibited the punctate distribution and overlapped with clathrin and transferrin, suggesting F13L was involved in the endocytosis of enveloped virus via clathrin-coated pits [36]. Further investigation on the association between VP19 protein with clathrin or other membrane structures will be helpful for understating the behaviors of VP19 at the early stage of virus infection. Differently, the expression of TFV ORF001, an orthologue of SGIV VP19 was mostly confined to the cytoplasm of the infected cells, especially around the viral assembly sites in the late stage of virus infection [21]. The difference of cellular localization might be due to the low identity (72%) between these two genes and the significant variations of their viral genome contents [14,37]. The translocation of VP19 into virus assembly sites was proposed to be accomplished with the help of other virus products. Another iridovirus core protein, RGV 53R has been demonstrated to play important roles during virus assembly and virion formation [10,29,32]. The detailed mechanism of VP19 in SGIV replication still remains to be determined.

## Conclusions

In conclusion, we cloned and characterized VP19 gene from SGIV. It encoded a cytoplasmic localized protein which was aggregated into the virus assembly site at the late stage of SGIV infection. Moreover, VP19 was ascertained as an envelope protein. Thus, we proposed that SGIV VP19 represented another conserved envelope protein associated with virus assembly in family *Iridoviridae*. Further studies will focus on the detailed function of this envelope protein during iridovirus life cycle.

## Methods

### Cells and virus

**Grouper spleen cells (GS) were maintained in** Leibovitz’s L-15 medium supplemented with 10% fetal bovine serum (FBS) (Gibco, USA) at 25°C [38]. Singapore grouper iridovirus (SGIV) used in this study was stored at −80°C until use.

### Bioinformatic analysis

The sequence of VP19 was used to analyze against the NCBI database using the WWW BLAST server (http://www.ncbi.nlm.nih.gov/blast). The motif contained in VP19 was predicted using MyHits web site (http://myhits.isb-sib.ch) [39]**.** Multiple amino acid sequences alignment was carried out using ClustalX 1.83 and edited with GeneDoc software. The conserved domain prediction was performed using SMART program (http://smart.embl-heidelberg.de/). The phylogenetic tree was established using the MEGA 4 software. GenBank accession numbers of VP19 homologs from different iridovirus for phylogenetic analysis were listed in Table 1.

**Table 1:** GenBank accession numbers of VP19 homologs used in this study

**Name of species (Abbreviation)**	**Accession number**
Singapore Groper Iridovirus (SGIV)	YP_164114
Groper iridovirus (GIV)	AAV91031
Common midwife toad ranavirus (CMTV)	AFA44906
European sheatfish virus (ESV)	YP_006347592
Epizootic haematopoietic necrosis virus (EHNV)	ACO25191
Rana grylio virus (RGV)	ABR08658
Frog virus 3 (FV3)	YP_031580
Tiger frog virus (TFV)	ABB92270
Ambystoma tigrinum virus (TFV)	YP_003772
Soft-shelled turtle iridovirus (STIV)	YP_002854233
Rock bream iridovirus (RBIV)	AAT71900
Infectious spleen and kidney necrosis virus (ISKNV)	AF371960
Wiseana iridescent virus (WSIV)	YP_004732814
Orange-spotted grouper iridovirus (OSGIV)	AY894343
Red sea bream iridovirus (RSIV)	BAK14314
Lymphocystis disease virus 1 (LCDV-1)	NP_078745
lymphocystis disease virus China (LCDV-C)	YP_073546
Insect iridescent virus 3 (IIV-3)	YP_654619
insect iridescent virus 6 (IIV-6)	NP_149800

### Plasmid construction, prokaryotic expression, and antibody preparation

To obtain the recombinant fusion protein, we amplified the truncate fragment of VP19 (VP19t) that without transmembrane domain using SGIV genome DNA as template. The expected fragment was subcloned into the prokaryotic expression vector pET-32a (Novagen, Germany) to obtain plasmid pET-VP19t. The full length of VP19 was amplified and subcloned into pEGFP-C1 vector to obtain plasmid EGFP-VP19. The recombinant plasmid was confirmed by DNA sequencing. The primer used in this study was listed in Table 2.

**Table 2:** Primers used in this study

**Primer name**	**Primer sequence (5′-3′)**
pET-VP19t-F	CGGAATTCATGGCATCGTCCACTATAC
pET-VP19t-R	GCCAAGCTTAATACTTTGAAAAATCGC
EGFP-VP19-F	CGCAAGCTTCGATGGCATCGTCCACTATAC
EGFP-VP19-R	AGTGGATCCCCCAAAAGAGCCCTTCATT
VP19-F	TCCAAGGGAGAAACTGTAAG
VP19-R	GGGGTAAGCGTGAAGACT
MCP-F	GCACGCTTCTCTCACCTTCA
MCP-R	AACGGCAACGGGAGCACTA
Actin-F	TACGAGCTGCCTGACGGACA
Actin-R	GGCTGTGATCTCCTTCTGCA

After IPTG induction of *E. coli* BL21 (DE3) containing pET-VP19t, the recombinant fusion protein rVP19t was purified using the HisBind purification kit (Novagen, Germany) according to the manufacture’s protocol. The purified protein was analyzed by sodium dodecyl sulfate-polyacrylamide gel electrophoresis (SDS-PAGE). Antibodies against rVP19t were prepared and its specificity was determined by western blotting as described as described previously [40].

### Membrane extraction

To determine whether VP19 was the component of envelope or capsid fraction, purified SGIV virus particles were treated with different detergents as described previously [10,30,31]. Briefly, purified virus were treated with 1% Triton-X 100 containing 150 mM NaCl at 37°C for 30 min, or treated with PBS containing 0.1% SDS at RT for 30 min. Then the mixture was centrifuged at 20 000 g for 30 min to separate the soluble and insoluble fraction. Proteins from the supernatant and pellet were electrophoresed by SDS-PAGE and transferred to PVDF membrane for western blotting analysis.

### Cell transfection and immune fluorescence assay

For transfection in vitro, cells were cultured into 24-well plates overnight. The plasmids, including pEGFP-C1 and EGFP-VP19 were transfected using Lipofectamine 2000 (invitrogen) according to the manufacturer’s instructions for further analysis.

To investigate the intracellular localization of VP19 during SGIV infection, immunofluorescence assay was carried out in GS cells infected with SGIV at MOI of 2. Briefly, at indicated time points, the coverslips were fixed with 4% paraformaldehyde and then blocked by 2% bovine serum albumin (BSA). After incubation with anti-VP19 serum (1:75) or negative serum (1:75), cells washed with PBS and incubated with FITC-conjugated goat anti-mouse antibodies (Pierce, USA) or Alexa Fluor® 555 Conjugated anti-mouse IgG (Cell signaling technology). Finally, cells were stained with 1 μg/ml 6-diamidino-2-pheny-lindole (DAPI), and observed under fluorescence microscopy (Leica, Germany).

### Western blotting

To detect the protein synthesis pattern during SGIV infection, GS infected with SGIV at MOI of 0.5 were collected at indicated time points as described above. Equal amounts of protein were subjected to SDS-PAGE and transferred to PVDF membranes. Membranes were blocked for 1 h with 5% non-fat dried milk solution and then incubated with the anti-VP19 serum or anti-MCP serum (1:2000) overnight at 4°C. After washing with TBST, the membranes were incubated for an additional hour with HRP-conjugated goat anti-mouse IgG at a dilution of 1:3000. Simultaneously, internal controls were detected by using antibody against β-actin. To examine the protein from CHX or Arac treated cells or detergent extracted virus particles, western blotting was carried out as described above.

### Electron microscopy

To detect the structure of virus, the purified SGIV virus particles and naked virions from 0.1% SDS-treated pellet were mounted on carbon-coated nickel grids (100 mesh) for 3 h at room temperature. After washing with PBS, the grids were stained with 2% phosphotungstic acid (PTA, pH 7.0) for 30 s. Specimens were examined under a transmission electron microscope (TEM) (JEM1400).

To determine the detailed localization of VP19 in virus particles, the purified SGIV virus particles and naked virions were mounted on grids as described above. Then the grids were blocked with 5% BSA for 2 h and then rinsed with PBS, followed by incubation with anti-VP19 (1:50) overnight at 4°C. The grids were washed with PBS, and then the sections were incubated with second antibody (1:50 anti-mouse goat IgG gold conjugate, 10 nm, Sigma) for 2 h at room temperature. Finally, the grids were washed with PBS and then stained with 2% PTA for 30 s. Specimens were observed as above. As a control, anti-MCP serum was used to detect the localization of capsid protein.

## Competing interests

The authors declared that they have no competing interests.

## Authors’ contributions

HXH and QQW designed and performed experiments, analyzed data and wrote the manuscript. GJ, OYZL, WSW and CXL performed the prokaryote expression, protein purification, Western blot assay and electron microscopy observation. HYH and QQW analyzed data and edited the manuscript. All authors read and approved the manuscript.
